# Different Role of Tumor Necrosis Factor-α Polymorphism in Non-Hodgkin Lymphomas among Caucasian and Asian Populations: A Meta-Analysis

**DOI:** 10.3390/ijms15057684

**Published:** 2014-05-05

**Authors:** Kan Zhai, Jie Ding, Yan Zhou

**Affiliations:** Medical Research Center, Beijing Chaoyang Hospital, Capital Medical University, Beijing 100020, China; E-Mails: ding201cy@sina.com (J.D.); abi373a@sohu.com (Y.Z.)

**Keywords:** TNF-α, polymorphism, NHL risk, meta-analysis, case-control

## Abstract

Tumor necrosis factor-α (TNF-α) is an immunoregulatory cytokine involved in B- and T-cell function, and also plays an important role in inflammation and cancer. TNF-α-308G>A has been associated with constitutively elevated TNF-α expression. Several studies have reported the association between the TNF-α-308G>A polymorphism and non-Hodgkin lymphomas (NHL) risk, however, results are still inconsistent. To solve these conflicts, we conducted the first meta-analysis to assess the effect of TNF-α-308G>A polymorphism on the risk of NHL and various subtypes (additive model) including 10,619 cases and 12,977 controls in Caucasian and Asian populations. Our meta-analysis indicated that TNF-α-308G>A polymorphism is not associated with NHL risk when pooling all studies together (OR = 1.06, 95% CI: 0.92–1.23, *p* = 0.413). In stratified analyses, we found TNF-α-308A allele was significantly associated with higher risk of NHL, B-cell lymphomas (BCL), T-cell lymphomas (TCL) and diffuse large B-cell lymphomas (DLBCL) in Caucasians (OR = 1.22, 95% CI: 1.06–1.40, *p* = 0.007; OR = 1.18, 95% CI: 1.03–1.34, *p* = 0.014; OR = 1.20, 95% CI: 1.01–1.42, *p* = 0.040; OR = 1.21, 95% CI: 1.11–1.32, *p* < 0.001, respectively). Interestingly, it was associated with decreased risk of NHL, BCL and DLBCL in Asians (OR = 0.75, 95% CI: 0.66–0.86, *p* < 0.001; OR = 0.70, 95% CI: 0.52–0.94, *p* = 0.018; OR = 0.70, 95% CI: 0.57–0.86, *p* = 0.001). These findings also suggest TNF-α might play a distinct role in pathogenesis of NHL in different populations.

## 1. Introduction

Non-Hodgkin lymphomas (NHL), a complex group of heterogeneous diseases of uncontrolled B- or T-cell proliferation with distinct clinical and histological features, accounts for approximately 90% of all malignancy lymphomas [1]. Malignant transformation of B- or T-cells can occur at different stages of maturation, which reflects the heterogeneity of malignancies with various biologic and clinical behaviors. B-cell lymphomas (BCL) comprise 90% of NHL. Diffuse large B-cell lymphomas (DLBCL) and follicular lymphomas (FL) are the two major subtypes of BCL. Clinical outcome of NHL varies from subtype, diagnosis and response to treatment, however, prognosis of T-cell lymphoma (TCL) is usually worse than that of BCL. Etiology of NHL is still poorly understood, although epidemiological studies have shown that individuals with innate or acquired immune deficiencies, immunosuppression and infection are at increased risk of NHL [2,3]. Recently, accumulating evidence has suggested that genetic variations such as single nucleotide polymorphisms (SNPs) are associated with NHL risk and survival [4–9]. Moreover, previous studies showing a 2- to 3-fold risk of NHL with a family history of hematological malignancies indicates that genetic factors might play a critical role in NHL pathogenesis [10–12].

Tumor necrosis factor-α (TNF-α) is one of the most important pro-inflammatory and tumor-related cytokines for its regulating immune response, inflammation, Th1/Th2 balance and lymphomagenesis [13]. Increased serum values of TNF-α have been detected in autoimmune disease and many malignancies including lymphomas [14–17]. TNF-α-308G>A (rs1800629) SNP has increased susceptibility to many kinds of tumors and autoimmune diseases, such as hepatocellular carcinoma, myeloma, lymphoma, ulcerative colitis, and Crohn’s disease [18–20]. TNF-α-308A allele is associated with higher constitutive and inducible TNF-α expression by affecting a consensus binding site of a transcription factor named activator protein-2 (AP-2) [21,22]. Studies using knockout mouse have supported that this cytokine could affect progression of BCL directly or indirectly [23,24]. Although the TNF-α-308G>A polymorphism has been widely assessed in association with NHL in different ethnicities, due to various sample sizes and genotyping methods, possibly because of NHL heterogeneity and other reasons, the results are still controversial.

In this study, we conducted the first comprehensive meta-analysis to test whether the TNF-α-308 polymorphism is associated with NHL overall risk or its subtypes, especially BCL, TCL, DLBCL, FL, chronic lymphocytic leukemia/small lymphocytic lymphomas (CLL/SLL), mantel cell lymphomas (MCL), mucosal-associated lymphomas (MALT), peripheral T-cell lymphomas (PTCL) and natural killer/T-cell lymphomas (NK/TCL). We also performed subgroup analysis by descent (Caucasians and Asians) to assess a possible factor that might influence the overall results. Therefore, this study might have more statistical power and increase precision to estimate association between TNF-α-308 polymorphism and its effect on NHL.

## 2. Results and Discussion

### 2.1. Eligible Studies

In the initial screening for key words, 405 potential articles were identified in PubMed, Embase and Cochrane Library. After removing duplication, 321 articles were needed for further assessment. Among them, 293 were excluded because of inappropriate study design or control samples. Of the remaining 28 relevant articles, 8 articles were excluded for using the same patients. 2 articles were also excluded for their controls in concordance with Hardy-Weinberg equilibrium (HWE). With strict including criteria, the final pool of eligible articles consisted of 18 articles involving a total of 10,619 patients with NHL and 12,977 healthy controls. Because of the large sample size, Caucasians and Asians were considered as population stratification in this meta-analysis. Table 1 shows characteristics of eligible articles including ethnicity, genotyping method, number of cases and controls and NHL pathological types. In fact, at the primary data extraction, allele frequencies in all controls of one study, which performed by Skibola, did not fulfill HWE [25]. This study is a meta- and pooled analysis adding more genotyping data to the initial pooled report [26] to confirm the association between TNF/LTA polymorphism and NHL risk in Caucasian and Asian populations. For the new subjects (including Caucasians and Asians) not included in the initial report (all were Caucasians) [26], allele frequency of TNF-α-308G>A in controls met HWE (*p* = 0.916). But TNF-α-308G>A in all controls in the initial report was not consistent with HWE (*p* = 0.0007). We analyzed the initial report composed of 8 subgroups comprehensively [26]. Finally, we excluded data of EPILYMPH-Spain, University of California San Francisco and the NCI-SEER Seattle subgroup, in which controls did not fulfill HWE, and extracted data successfully. Since Skibola *et al.* [25] conducted this large pooled analysis in TNF polymorphism on NHL risk in Caucasians and Asians, we separated this paper into two studies according to population. In addition, 13 studies were conducted on Caucasians [16,27–38], and 4 were on Asians [39–42]. Several genotyping methods were used, including allelic specific polymerase chain reaction (ASPCR), polymerase chain reaction-restriction fragment length polymorphism (PCR-RFLP), polymerase chain reaction-solid-phase minisequencing (PCR-SPM), polymerase chain reaction-ligation detection reaction (PCR-LDR), TaqMan, Sequenom and sequencing.

### 2.2. Quantitative Synthesis

Based on a large pooled sample size, we analyzed TNF-α-308G>A polymorphism effects on risks of NHL, BCL, TCL and subtypes (DLBCL, FL, CLL/SLL, MCL, MALT, PTCL and NK/TCL) in additive model (A *vs.* G) which stratified by ethnicity (Caucasians and Asians). Results of meta-analysis and primary data extracted from studies are listed in Tables 2 and 3.

#### 2.2.1. TNF-α-308G>A and NHL

**Figure 1.**
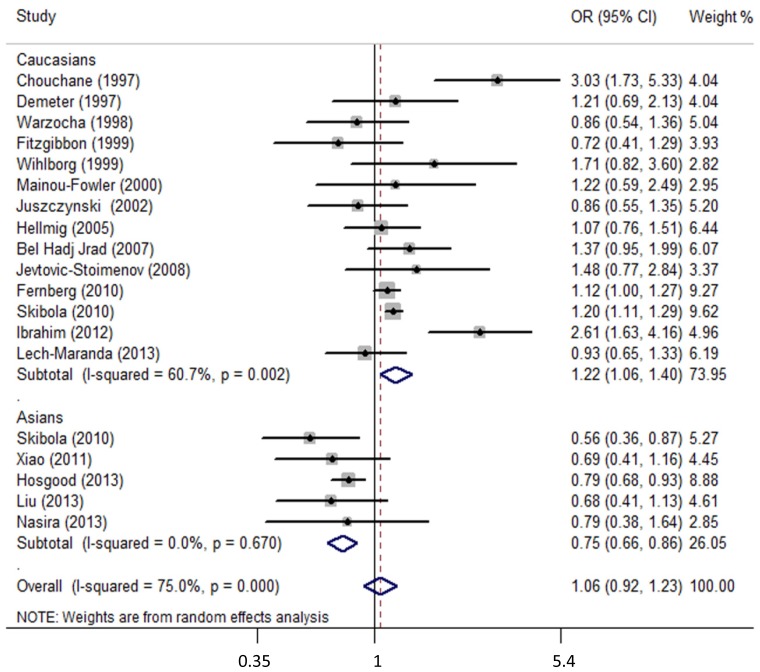
Overall association between TNF-α-308G>A polymorphism and NHL risk (additive model) in Caucasian and Asian populations. For each study, the estimate of odds ratio (OR) and its 95% confidence interval (CI) is plotted with a box and a horizontal line. The symbol diamond indicates pooled OR and its 95% CI.

Of the combined 10,619 patients with NHL and 12,977 controls, no risk association was observed in TNF-308G>A polymorphism and NHL with significant heterogeneity between studies (OR = 1.06, 95% CI: 0.92–1.23, *p* = 0.413; I^2^ = 75.0%, *p*_het_ < 0.001). In subgroup analysis based on population, significant associations were detected in Caucasian and Asian populations, respectively. In Caucasians with 7893 cases and 8447 controls, participants with TNF-308A allele had an increased NHL risk (OR = 1.22, 95% CI: 1.06–1.40, *p* = 0.007; I^2^ = 60.7%, *p*_het_ = 0.002). However, in Asians with 2726 cases and 4530 controls, decreased risk was observed (OR = 0.75, 95% CI: 0.66–0.86, *p* < 0.001; I^2^ = 0.0%, *p*_het_ = 0.670). Figure 1 shows the forest plot of the overall association between TNF-α-308G>A polymorphism and NHL risk in additive model (A *vs.* G) stratified by ethnicity.

To evaluate the influence of each study on the pooled ORs in two subgroups, we deleted single study at a time to recalculate the influence of individual study for the outcome of the meta-analysis. The pooled ORs were stable and in an effective interval with statistical significant though the fixed-effect in additive model estimating before or after any single study deleted in each group (data not shown). These indicated that the results of this meta-analysis were reliable and had not been overly influenced by any one of studies.

**Figure 2.**
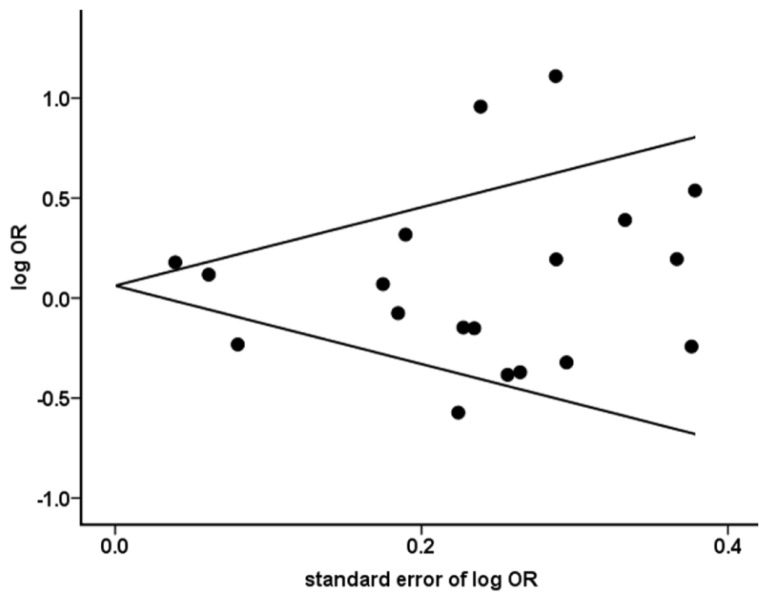
Begg’s funnel plot for publication bias on the association between TNF-α-308G>A polymorphism and NHL risk in additive model.

We performed Begg’s funnel plot and Egger’s test to evaluate the publication bias of all included studies. Figure 2 shows no evidence of obvious asymmetry in overall analysis for TNF-α-308G>A polymorphism in additive model (*p*_Begg’s_ = 0.529). Egger’s test also suggested no significant publication bias existed in this meta-analysis (additive model, *p* = 0.780).

#### 2.2.2. TNF-α-308G>A and B- or T-CL

Thirteen studies comprising a total of 20,064 participants (8092 cases with BCL and 11,972 controls) and 4 studies including 11,832 participants (1100 cases with TCL and 10,732 controls) were analyzed for an association between TNF-α-308G>A polymorphism and BCL or TCL risk.

For BCL analysis, the pooled OR across all studies was not statistically significant (OR = 1.07, 95% CI: 0.91–1.26, *p* = 0.411; I^2^ = 74.4%, *p*_het_ < 0.001). Increased risk was found in Caucasians (OR = 1.18, 95% CI: 1.03–1.34, *p* = 0.014; I^2^ = 44.6%, *p*_het_ = 0.054), decreased risk was detected in Asians (OR = 0.70, 95% CI: 0.52–0.94, *p* = 0.018; I^2^ = 41.9%, *p*_het_ = 0.189). In TCL analysis, TNF-308A associated with a higher NHL risk in Caucasians (OR = 1.20, 95% CI: 1.01–1.42, *p* = 0.040; I^2^ = 0.0%, *p*_het_ = 0.361).

#### 2.2.3. TNF-α-308G>A and NHL Subtypes

For DLBCL, the most common NHL subtype, there were 3353 cases and 10,817 controls included in the analysis. Consistent with the results for all NHL, the TNF-α-308A allele was associated with an increased risk of DLBCL in Caucasians (OR = 1.21, 95% CI: 1.11–1.32, *p* < 0.001; I^2^ = 0.0%, *p*_het_ = 0.491), but with a decreased risk in Asians (OR = 0.70, 95% CI: 0.57–0.86, *p* = 0.001; I^2^ = 0.0%, *p*_het_ = 0.908). The pooled OR was 0.97 (95% CI: 0.75–1.26, *p* = 0.840; I^2^ = 83.9%, *p*_het_ < 0.001).

No associations were found in TNF-α-308G>A polymorphism with FL, CLL/SLL, MCL, MALT, PTCL and NK/TCL in overall and each ethnic subgroup.

### 2.3. Discussion

In the pooled analysis of 18 articles, we found TNF-α-308G>A polymorphism to be significantly associated with NHL risk in Caucasians and Asians. We provided evidence that subjects with TNF-α-308A allele had an increased risk of NHL in Caucasians, and had a decreased risk in Asians. Similar results were confirmed in analyses of BCL and DLBCL. Further, the TNF-α-308A allele was positively associated with risks of TCL in Caucasians. Our study highlights the effect of TNF-α gene polymorphism on risks of NHL and its subtypes in different populations. These findings indicate a potential connection between constitutively higher TNF-α expression and pathogenesis of NHL.

TNF-α is a transmembrane protein and mainly produced by macrophages and is expressed at low levels in a wide variety of cells. TNF-α mediates its effects through TNF-α receptor 1 and 2 (TNFR1 and TNFR2) by ligand passing and signal transduction. TNFR1 has a death domain that could interact with TNF-α receptor-associated death domain (TRADD), sequentially recruiting proteins to induce caspase-3 activation for apoptosis. TRADD could also bind TNF receptor-associated factor 2 (TRAF2) to recruits proteins activating IKK, GCK, and RIP, which finally leads to the NF-κB, JNK, and MAPK pathway activation for anti-apoptosis and cell survival. Although TNFR2 lacks the death domain, it could also bind TRAF2 to active an anti-apoptosis pathway [13,43]. Aberrant NF-κB activation is a hallmark of several lymphomas for promoting continuous lymphocyte proliferation, which is also directly linked to disease promotion [44,45]. When cells are exposed to TNF-α, NF-κB pathway activation leads to the expression of many genes to cause chronic inflammation, which stimulates tumor growth. Dysregulated TNF-α contributes directly to the transformed state in many cancers, especially those of BCL [46]. Collectively, TNF-α acting as an immunoregulatory cytokine builds a bridge between inflammation and cancer by activating many biological pathways including the nuclear factor-κB (NF-κB) pathway in promoting cell proliferation, survival, transformation, invasion and angiogenesis.

Previous studies suggest higher expression of TNF-α is associated with NHL risk at the time of diagnosis [14–17]. These studies do not contradict the results of TNF-α-308A allele inducing higher constitutively TNF-α expression associated with decreased NHL risk in Asians. With a heterogeneous malignancy and population diversity, we believe the level of constitutively TNF-α expression must play a different vital role at the step of NHL initiation in Caucasians and Asians, although the reasons for this have not been understood. Once a tumor forms, it secretes TNF-α to promote its survival, proliferation and metastasis. A subtype of TCL failing to express TNF-α and frequently with the TNF-α gene promoter methylated [47] indicates that epigenetic changes might also influence NHL susceptibility together. Environmental, occupational exposure and pathogenic agent infection (such as Epstein-Barr virus and human T-cell leukemia virus-1) are the well-known risk factors for NHL [48,49]. Therefore, genetic, epigenetic, tumor microenvironment, environment and their interaction could together contribute to NHL progression. Few studies about this kind of interaction relative to NHL susceptibility have been published. Due to insufficient data, our meta-analysis did not combine the effects of these factors in an association analysis between genetic variation and NHL risk. Much more precise investigations should be performed to clarify the true association of these types of interactions with polymorphism and NHL.

TNF-α and LT-α gene lie in the major histocompatibility complex class III, telemetric to the class II and centrometric to class I gene. Therefore, TNF-α being in linkage disequilibrium (LD) with these genes may also be linked to another region, haplotype or extended, that can influence NHL development [50]. This meta-analysis only evaluated one SNP in the TNF-α gene, though it was not possible to analyze haplotypes with the present data. Further studies will be needed to pool data and analyze whether haplotypes comprising TNF-α-308G>A and other SNPs are linked to NHL risk, and clarify their function concomitantly.

Because of relatively low incidence of NHL, sample sizes of some studies included in this analysis are very small. The major strength of this study is the larger pooled sample size involving a total of 10,619 patients with NHL and 12,977 healthy controls for association study, which would largely minimize the possibility of chance findings. In addition, patients in our study were all Caucasians or Asians, which would exclude the biased results due to population stratification.

In conclusion, we performed the first comprehensive meta-analysis involving 10,619 patients with NHL and 12,977 controls from 18 articles to evaluate the association between TNF-α-308G>A polymorphism and NHL risk. Our study showed that TNF-α-308G>A SNP in the promoter region of TNF-α gene is associated with NHL risk. In addition, TNF-α-308A increases risks of NHL, BCL, TCL and DLBCL in the Caucasian population; however, interestingly, it reduces risks of NHL, BCL and DLBCL in the Asian population. This association might be mediated by constitutive changes of TNF-α expression in individuals carrying the -308A allele, to induce inflammatory responses or the alternative pathway which be involved in NHL initiation and progression. Our meta-analysis emphasizes that genetic variation plays a crucial role in cancer; theTNF-α-308G>A polymorphism might play a role in a specific subtype of NHL and its importance varies in different populations. Further studies should focus on the function of how variants affect NHL in different populations and the elucidation of the pathway involved which may eventually lead to a better understanding of tumorigenesis and contribute to the prevention of NHL.

## 3. Experimental Section

### 3.1. Publication Search

We carried out a search in three electronic databases PubMed, Embase and Cochrane Library to find relevant publications up to November 2013, using key words related to the TNF-α gene polymorphism in combination with various NHL subtypes [51]. The search was limited to studies that had been conducted on human subjects and without language restriction. Reference lists of the retrieved articles, reviews and editorials were also screened to find all additional eligible studies.

### 3.2. Inclusion Criteria

Selection of studies had to meet the following criteria: (1) case-control studies, family or sibling pairs studies were excluded; (2) published in English; (3) subjects were limited to adult and without autoimmune diseases, studies with children were also excluded; (4) DNA was extracted from peripheral blood leukocytes; (5) study described the association between TNF-α-308 polymorphism and NHL risk; (6) sufficient data for estimating odds ratio (OR) and its corresponding 95% confidence interval (95% CI); (7) control group fulfilled HWE. When the same subject group occurred in more than one study, only the complete study was chosen to be included in this meta-analysis.

### 3.3. Data Extraction

An initial screening of title and abstract was performed for the first step, followed by further screening based on full-text review. Information was independently extracted from all eligible publications by two investigators (K.Z. and J.D.), including the first author, publication year, ethnicity, sample size, genotyping method, the number of each genotype in cases and controls. For studies with subjects of different ethnic groups and had sufficient information, we extracted data separately for each ethnicity. Disagreements were resolved through discussion.

### 3.4. Statistical Analysis

We assessed the association between TNF-α-308G>A polymorphism and NHL risk by crude ORs and 95% CIs in an additive model. Heterogeneity among studies was examined with I^2^ statistics. In this meta-analysis, I^2^ > 50% was defined as heterogeneity. Fixed-effect model (Mantel-Haenszel method) was used to evaluate inter-study heterogeneity. If heterogeneity existed, random-effect model (DerSimonian-Laird method) was used. *Z* test was used to determine the pooled OR and 95% CI. Analyses were also conducted on the subgroups of studies based on ethnicity. The potential influence of publication bias was assessed using Begg’s funnel plot and Egger’s linear regression test [52,53]. To evaluate the effect of one single study on overall risk of NHL, sensitivity analyses by excluding every study and recalculating ORs and 95% CI were conducted [54]. HWE in controls of each study was examined by the Pearson’s goodness-of-fit χ^2^ test. All statistical tests were carried out with SPSS 16.0 (SPSS Inc., Chicago, IL, USA) and Stata 12.0 (StataCorp, College Station, TX, USA). A 2-tailed *p* < 0.05 was considered as statistical significance.

## 4. Conclusions

We performed the first comprehensive meta-analysis involving 10,619 patients with NHL and 12,977 controls from 18 articles to evaluate the association between TNF-α-308G>A polymorphism and NHL risk. Our study showed that TNF-α-308G>A SNP in the promoter region of TNF-α gene is associated with NHL risk. In addition, TNF-α-308A increases risks of NHL, BCL, TCL and DLBCL in the Caucasian population; interestingly, this polymorhism reduces risks of NHL, BCL and DLBCL in the Asian population. This association might be mediated by constitutive changes of TNF-α expression in individuals carrying the -308A allele, to induce inflammatory responses or the alternative pathway which be involved in NHL initiation and progression. Our meta-analysis emphasizes that genetic variation plays a crucial role in cancer; the TNF-α-308G>A polymorphism might play a role in a specific subtype of NHL and its importance may vary in different populations. Further studies should be focused on how variants affect NHL in different populations and the elucidation of the pathways involved which may eventually lead to a better understanding of tumorigenesis and contribute to the prevention of NHL.

## Figures and Tables

**Table 1.** Characteristics of 18 eligible articles included in this meta-analysis.

Study	Ethnicity	Genotyping Method	Samples		Characteristics

			NHLs (n)	Controls (n)	
Chouchane, 1997	Caucasians	ASPCR	44	106	All subtypes
Demeter, 1997	Caucasians	PCR-RFLP	63	117	HCL only
Warzocha, 1998	Caucasians	ASPCR	273	96	All subtypes
Fitzgibbon, 1999	Caucasians	PCR-RFLP	121	88	FL only
Wihlborg, 1999	Caucasians	PCR-SPM	49	51	CLL only
Mainou-Fowler, 2000	Caucasians	PCR-RFLP	76	40	CLL
Juszczynski, 2002	Caucasians	Sequencing	204	120	All subtypes
Hellmig, 2005	Caucasians	TaqMan	138	533	MALT only
Bel Hadj Jrad, 2007	Caucasians	PCR-RFLP	194	160	All subtypes
Jevtovic-Stoimenov, 2008	Caucasians	PCR-RFLP	80	34	All subtypes
Fernberg, 2010	Caucasians	Sequenom	2267	1484	All subtypes
Skibola, 2010	Caucasians, Asians	TaqMan or Pyrosequencing	4287	5591	All subtypes
Xiao, 2011	Asians	PCR-RFLP	160	214	All subtypes
Ibrahim, 2012	Caucasians	PCR-RFLP	84	100	BCL only
Hosgood, 2013	Asians	TaqMan	291	300	All subtypes
Lech-Maranda, 2013	Caucasians	TaqMan	288	192	CLL only
Liu, 2013	Asians	PCR-LDR	1932	3622	TCL only
Nasira, 2013	Asians	PCR-RFLP	68	129	All subtypes

Abbreviations: NHL, non-Hodgkin lymphomas; ASPCR, allelic specific polymerase chain reaction; PCR-RFLP, polymerase chain reaction-restriction fragment length polymorphism; PCR-SPM, polymerase chain reaction-solid-phase minisequencing; PCR-LDR, polymerase chain reaction-ligation detection reaction; HCL, hairy cell leukemias; FL, follicular lymphomas; CLL, chronic lymphocytic leukemias; MALT, mucosal-associated lymphomas; BCL, B-cell lymphomas; TCL, T-cell lymphomas.

**Table 2.** Stratified analyses of TNF-α-308G/A polymorphism on NHL risk in Caucasians and Asians *.

Type	Ethnicity	Study (n)	Samples		OR (95% CI)	*p*	I^2^ (%)	*p*_het_

			Cases (n)	Controls (n)				
NHL	Caucasians	14	7893	8447	1.22 (1.06–1.40)	0.007	60.7	0.002
	Asians	5	2726	4530	0.75 (0.66–0.86)	<0.001	0.0	0.670
	Overall	19	10,619	12,977	1.06 (0.92–1.23)	0.413	75.0	<0.001

BCL	Caucasians	11	6369	8085	1.18 (1.03–1.34)	0.014	44.6	0.054
	Asians	2	1723	3887	0.70 (0.52–0.94)	0.018	41.9	0.189
	Overall	13	8092	11,972	1.07 (0.91–1.26)	0.411	74.4	<0.001

TCL	Caucasians	2	467	6810	1.20 (1.01–1.42)	0.040	0.0	0.361
	Asians	2	633	3922	0.96 (0.74–1.23)	0.723	57.8	0.124
	Overall	4	1100	10,732	1.11 (0.96–1.28)	0.145	43.1	0.153

DLBCL	Caucasians	3	2325	6930	1.21 (1.11–1.32)	<0.001	0.0	0.491
	Asians	2	1028	3887	0.70 (0.57–0.86)	0.001	0.0	0.908
	Overall	5	3353	10,817	0.97 (0.75–1.26)	0.840	83.9	<0.001

FL	Caucasians	3	1233	6898	1.00 (0.89–1.13)	0.949	31.0	0.235
	Asians	2	184	3887	0.72 (0.47–1.12)	0.142	12.4	0.285
	Overall	5	1417	10,785	0.98 (0.87–1.10)	0.706	32.3	0.206

CLL/SLL	Caucasians	6	1859	7127	1.02 (0.92–1.13)	0.767	13.8	0.326

MCL	Caucasians	2	250	6810	1.25 (1.00–1.57)	0.052	0.0	0.560

MALT	Caucasians	1	138	533	1.07 (0.76–1.51)	0.689		

PTCL	Caucasians	1	183	5326	1.11 (0.85–1.47)	0.446		
	Asians	1	79	300	0.65 (0.29–1.48)	0.303		
	Overall	2	262	5636	1.05 (0.80–1.36)	0.741	32.9	0.222

NK/TCL	Asians	2	190	3922	0.74 (0.46–1.17)	0.196	12.4	0.285

* Fixed-effect model was used when *p* value for heterogeneity test >0.05; otherwise, random-effect model was used;

Abbreviations: TNF, tumor necrosis factor; NHL, non-Hodgkin lymphomas; BCL, B-cell lymphomas; TCL, T-cell lymphomas; DLBCL, diffuse large B-cell lymphomas; FL, follicular lymphomas; CLL/SLL, chronic lymphocytic leukemias/small lymphocytic lymphomas; MCL, mantel cell lymphomas; MALT, mucosal-associated lymphomas; PTCL, peripheral T-cell lymphomas; NK/TCL, natural killer/T-cell lymphomas.

**Table 3.** Summary of primary data from eligible studies in this meta-analysis.

Type	Ethnicity	Study	Cases			Controls		
	
			GG	GA	AA	GG	GA	AA
NHL	Caucasians	Chouchane, 1997	11	33	0	72	33	1
		Demeter, 1997	42	18	3	81	34	2
		Warzocha, 1998	203	65	5	69	24	3
		Fitzgibbon, 1999	96	23	2	64	22	2
		Wihlborg, 1999	29	19	1	37	14	0
		Mainou-Fowler, 2000	50	23	3	28	11	1
		Juszczynski, 2002	151	49	4	85	32	3
		Hellmig, 2005	93	39	6	360	160	13
		Bel Hadj Jrad, 2007	120	59	15	107	49	4
		Jevtovic-Stoimenov, 2008	32	46	2	19	14	1
		Fernberg, 2010	1490	675	102	1007	431	46
		Skibola, 2010	2712	1136	164	3791	1394	141
		Ibrahim, 2012	41	21	22	67	27	6
		Lech-Maranda, 2013	213	67	8	136	53	3

	Asians	Skibola, 2010	243	29	3	212	49	4
		Xiao, 2011	138	20	2	174	35	5
		Hosgood, 2013	1702	221	9	3091	506	25
		Liu, 2013	264	27	0	260	40	0
		Nasira, 2013	58	9	1	105	22	2

BCL	Caucasians	Demeter, 1997	42	18	3	81	34	2
		Fitzgibbon, 1999	96	23	2	64	22	2
		Wihlborg, 1999	29	19	1	37	14	0
		Mainou-Fowler, 2000	50	23	3	28	11	1
		Juszczynski, 2002	72	29	3	85	32	3
		Hellmig, 2005	93	39	6	360	160	13
		Jevtovic-Stoimenov, 2008	24	29	2	19	14	1
		Fernberg, 2010	1395	630	91	1007	431	46
		Skibola, 2010	2221	915	139	3791	1394	141
		Ibrahim, 2012	41	21	22	67	27	6
		Lech-Maranda, 2013	213	67	8	136	53	3

BCL	Asians	Skibola, 2010	194	23	2	212	49	4
		Hosgood, 2013	1332	164	8	3091	506	25

TCL	Caucasians	Fernberg, 2010	95	45	11	1007	431	46
		Skibola, 2010	216	90	10	3791	1394	141

	Asians	Hosgood, 2013	287	54	1	3091	506	25
		Liu, 2013	264	27	0	260	40	0

DLBCL	Caucasians	Fernberg, 2010	371	173	23	1007	431	46
		Juszczynski, 2002	72	29	3	85	32	3
		Skibola, 2010	1093	495	66	3791	1394	141

	Asians	Skibola, 2010	86	11	2	212	49	4
		Hosgood, 2013	829	97	3	3091	506	25

FL	Caucasians	Fitzgibbon, 1999	96	23	2	64	22	2
		Fernberg, 2010	297	115	12	1007	431	46
		Skibola, 2010	489	167	32	3791	1394	141

	Asians	Skibola, 2010	48	6	0	212	49	4
		Hosgood, 2013	115	13	2	3091	506	25

CLL/SLL	Caucasians	Wihlborg, 1999	29	19	1	37	14	0
		Mainou-Fowler, 2000	50	23	3	28	11	1
		Jevtovic-Stoimenov, 2008	24	29	2	19	14	1
		Fernberg, 2010	373	171	24	1007	431	46
		Skibola, 2010	605	193	25	3791	1394	141
		Lech-Maranda, 2013	213	67	8	136	53	3

MCL	Caucasians	Fernberg, 2010	76	33	10	1007	431	46
		Skibola, 2010	90	35	6	3791	1394	141

MALT	Caucasians	Hellming, 2005	93	39	6	360	160	13

PTCL	Caucasians	Skibola, 2010	125	53	5	3791	1394	141

	Asians	Liu, 2013	72	7	0	260	40	0

NK/TCL	Asians	Hosgood, 2013	89	14	0	3091	506	25
		Liu, 2013	81	6	0	260	40	0

Abbreviations: NHL, non-Hodgkin lymphomas; BCL, B-cell lymphomas; TCL, T-cell lymphomas; DLBCL, diffuse large B-cell lymphomas; FL, follicular lymphomas; CLL/SLL, chronic lymphocytic leukemias/small lymphocytic lymphomas; MCL, mantle cell lymphomas; MALT, mucosal-associated lymphomas; PTCL, peripheral T-cell lymphomas; NK/TCL, NK/T-cell lymphomas.

## Abbreviations

TNF: tumor necrosis factor
NHL: non-Hodgkin lymphomas
BCL: B-cell lymphomas
TCL: T-cell lymphomas
DLBCL: diffuse large B-cell lymphomas
FL: follicular lymphomas
CLL/SLL: chronic lymphocytic leukemias/small lymphocytic lymphomas
MCL: mantel cell lymphomas
MALT: mucosal-associated lymphomas
PTCL: peripheral T-cell lymphomas
NK/TCL: natural killer/T-cell lymphomas
OR: odds ratio
95% CI: 95% confidence interval
